# A multi-epitope vaccine GILE against *Echinococcus Multilocularis* infection in mice

**DOI:** 10.3389/fimmu.2022.1091004

**Published:** 2023-01-17

**Authors:** Pei Zhou, Zhen Zhou, Meiduo Huayu, Lei Wang, Lin Feng, Yang Xiao, Yao Dai, Mingyuan Xin, Feng Tang, Runle Li

**Affiliations:** ^1^ Qinghai University Medical College, Xining, Qinghai, China; ^2^ Research Center for High Altitude Medicine, Qinghai-Utah Joint Research Key Lab for High Altitude Medicine, Qinghai Provincial Key Laboratory of Plateau Medical Application, Key Laboratory of Ministry of Education, Qinghai University, Xining, Qinghai, China; ^3^ Department of Pathology, The Second Xiangya Hospital DE Central South University, Changsha, Hunan, China

**Keywords:** *Echinococcus multilocularis*, multi-epitope vaccine, prokaryotic expression, immune, bioinformatics

## Abstract

**Introduction:**

The objective of this study is to construct a multi-epitope vaccine GILE containing B-cell and T-cell epitopes against *Echinococcus Multilocularis* (*E. multilocularis*) infection based on the dominant epitopes of *E. multilocularis* EMY162, LAP, and GLUT1.

**Methods:**

The structure and hydrophobicity of GILE were predicted by SWISSMODEL, pyMOL, SOPMA and VMD, and its sequence was optimized by Optimum™ Codon. The GILE gene was inserted into pCzn1 and transformed into *Escherichia coli* Arctic express competent cells. IPTG was added to induce the expression of recombinant proteins. High-purity GILE recombinant protein was obtained by Ni-NTA Resin. BALB/c mice were immunized with GILE mixed with Freund’s adjuvant, and the antibody levels and dynamic changes in the serum were detected by ELISA. Lymphocyte proliferation was detected by MTS. The levels of IFN-g and IL-4 were detected by ELISpot and flow cytometry (FCM). T cells were detected by FCM. The growth of hepatic cysts was evaluated by Ultrasound and their weights were measured to evaluate the immune protective effect of GILE.

**Results:**

The SWISS-MODEL analysis showed that the optimal model was EMY162 _95-104_―LAP_464-479_―LAP_495-510_―LAP_396-410_―LAP_504-518_―EMY162_112-126_. The SOPMA results showed that there were Alpha helix (14.88%), Extended strand (26.25%), Beta turn (3.73%) and Random coil (45.82%) in the secondary structure of GILE. The restriction enzyme digestion and sequencing results suggested that the plasmid pCzn1-GILE was successfully constructed. The SDSPAGE results indicated that the recombinant protein was 44.68 KD. The ELISA results indicated that mice immunized with GILE showed higher levels of serum antibodies compared to the PBS group. The FCM and ELISpot results indicated that mice immunized with GILE secreted more IFN-g and IL-4. Immunization with GILE also led to a significant decrease in the maximum diameter and weight of cysts and stimulated the production of CD4^+^ and CD8^+^ T Cell.

**Discussion:**

A multi-epitope vaccine GILE with good immunogenicity and antigenicity has been successfully constructed in this study, which may provide important theoretical and experimental bases for the prevention and treatment of *E. multilocularis* infection.

## 1 Introduction

Alveolar echinococcosis (AE) is a parasitic disease that is prevalent throughout the world except in Antarctica, and it is most prevalent in the northern hemisphere, especially in developing countries such as China, Russia, Kazakhstan and Mongolia (1). The incidence of AE is reported to be about 18,200 cases per year worldwide, resulting in about 666,000 disability adjusted life years, and it is important to point out that 91% of new cases occur in China (2). Human is an accidental intermediate host of *Echinococcus multilocularis* (*E. multilocularis*). Once the eggs are ingested, they will hatch into oncosphere larvae in the intestine and then migrate into tissues and organs of the host (3). Patients infected with *E. multilocularis* can present with diverse clinical symptoms, including liver dysfunction, ascites, pulmonary insufficiency, and neurological disorders (4), but there are no specific symptoms for the diagnosis of AE (5). It is noteworthy that AE is difficult to treat because *E. multilocularis* grows aggressively like a malignant tumor (6). Surgery is the main therapeutic option, but its outcomes are compromised by postoperative complications. The use of broad-spectrum anthelmintics such as albendazole is also associated with poor outcomes. Because of this, the current cure rate of AE is only 30-40% per year (7). Recent studies have suggested that vaccination is an effective means for the control and prevention of *E. multilocularis* infection (8), and immunization with recombinant proteins of parasites, such as leucine aminopeptidase (LAP), EMY162, EM95 and Tetraspanin (TSP), could protect against *E. multilocularis* infection and infiltration in host livers (9). EMY162 and EM95 are secreted proteins expressed in all life stages of *E. multilocularis* (10); and TSP plays an important role in the growth and development of *E. multilocularis* (11). EM14-3-3 is also closely related to the growth and development of *E. multilocularis* and thus could be a candidate vaccine for *E. multilocularis.* Our animal experiments have demonstrated that EM14-3-3 has two dominant antigen epitopes of B cells, two Th1 dominant antigen epitopes, and one Th2 dominant antigen epitope. This work provides a basis for the development of effective and safe vaccines targeting the epitopes of *E. multilocularis* (12, 13).However, the use of a single epitope vaccine might not be adequate to prevent *E. multilocularis* infection because different types and amounts of proteins would be expressed at different developmental stages of *E. multilocularis* (14). In this study, a multi-epitope vaccine GILE was designed using bioinformatics and molecular biology techniques based on the dominant epitopes of *E. multilocularis* EMY162, LAP and GLUT1 (EmEMY162, EmLAP, and EmGLUT1) identified in our previous studies (9, 10, 15), and its immune protective effect against *E. multilocularis* infection was evaluated in mice.

In this study, we evaluated the immunogenicity and immune protection of GILE. The size of cyst was measured by *in vitro* ultrasound to evaluate the effect of GILE. The level of antibody, cytokines and CD4^+^ T cell and CD8^+^ T cell were detected by Elisa, Elispot, FCM (flow cytometry), so as to evaluate the effectiveness of the immune response. Finally, the protective effect of the vaccine was evaluated comprehensively.

## 2 Materials and methods

### 2.1 Bioinformatics analysis

The protein amino acid sequences of EmEMY162, EmLAP, and EmGLUT1 were obtained from National Centre for Biotechnology Information (NCBI) (https://www.ncbi.nlm.nih.gov/protein) in FASTA format. T-cell and B-cell dominant epitopes were predicted using IEDB online software (http://tools.immuneepitope.org/main/), based on which a multi-epitope vaccine was constructed. B cell and Th cell epitopes were joined together by KK and GS linkers, respectively. The gene sequence of GILE was designed by online bioinformatics tools (Uniport, PDB). The secondary and tertiary structure was predicted online using SOPMA and SWISS-MODEL, respectively. The hydropathicity was predicted using VMD (15).

### 2.2 Prokaryotic expression and purification of proteins

The expression of GILE proteins was determined as described previously (16). The GILE gene was connected to Nde I and Xba I of pCzn1 vector by double enzyme digestion to obtain the expression plasmid pCzn1-GILE (17), which was transfected into *Escherichia coli* Arctic express competent cells to express GILE proteins. After IPTG induction at 37°C for 4 h, the supernatant was removed by centrifugation at 10000 r/min and 4°C for 3 min, and cells were suspended in 1 × PBS (pH=8.0). After that, cells were crushed in an ultrasonic ice bath, and GILE inclusion bodies were dissolved in 8 M urea in 1 × PBS (pH=8.0). GILE was purified by high-affinity Ni-NTA Resin. After concentration in polyethylene glycol 20000 (PEG20000), the purified target proteins were subjected to 12% SDS-PAGE and Western blot analysis (12) and stored at −80°C.

### 2.3 Immunogenicity and immune protection experiments

All animal experiments were performed in compliance with the regulations of the Ministry of Science and Technology of China and approved by the Experimental Committee of Qinghai University (QHDX-2018-09). Six to eight-week-old male BALB/c specific pathogen free (SPF) mice were purchased from Beijing Spaefer Biotechnology Company (SCXK2019-0010) and fed with sterilized food and water over the 24-h day/night cycle in the Animal Biosafety Level II Laboratory (ABSL-2) of the Research Center for High Altitude Medicine of Qinghai University.

Immunogenicity experiments were performed as described previously (18). Briefly, mice were randomly divided into two (GILE and PBS) groups of 6 mice each and injected intraperitoneally with GILE or PBS mixed with an equal volume of Freund’s adjuvant (50 μg/mouse) once a week for 4 weeks. Complete Freund’s adjuvant (CFA) was used for the primary immunization; incomplete Freund’s adjuvant (IFA) was used for the second and third booster immunization; and pure protein was used for the last immunization. During immunization, blood samples were collected from the tail vein every other day to monitor dynamic changes of antibodies. Two weeks after the last immunization, mice were sacrificed and their serum and splenocytes were harvested for FCM, MTS, ELISA and ELISPOT tests.

Immune protection experiments were performed as described previously with some minor modifications (10). Mice were randomly divided into 4 groups (1): Polypeptide vaccine (GILE) group (n=6). GILE was thoroughly mixed with an equal volume of Freund’s adjuvant and intraperitoneally injected (50 μg/mouse) once a week for 4 weeks (2); Protein (LAP, EMY162, and GLEP (polypeptide recomposed of GLUT1 sequences)) groups (n=6). Mice were immunized with LAP, GLEP, or EMY162 proteins following the procedure described above (3); Epitope (LAP_464-479_, LAP_495-510_, EMY162 _95-108_, and EMY162 _112-126_) groups (n=6). Mice were immunized with LAP_464-479_, LAP_495-510_, EMY162 _95-108_, or EMY162 _112-126_ epitope peptides following the procedure described above (4); Non-immunized (PBS) group (n=6). Mice was intraperitoneally injected with PBS mixed with an equal volume of Freund’s adjuvant following the procedure described above.

Two weeks after the fourth immunization, the infection model was established with *E. multilocularis* protoscoleces obtained from the Basic Immunology Laboratory for Zoonosis of Qinghai University. Protoscoleces were counted and diluted to 5 per/μL, and 200 μL of the mixture containing 1000 protoscoleces were injected into the abdominal cavity of immunized mice. After four months, mice were sacrificed to evaluate the protective effect of vaccine.

### 2.4 Detection of antibodies

Serum antibodies were determined by indirect ELISA. Briefly, GILE, LAP, GLEP, EMY162, LAP_464-479_, LAP_495-510_, EMY162 _95-108_, EMY162 _112-126_, and GLUT_424-432_ (10 μg/mL) were respectively coated on ELISA plates overnight at 4 °C. The plates were washed three times with PBST and blocked with 3% Al-bumin Bovine V, and serum (1: 3000) was added (100 μL/well) and incubated at 37°C for 1 h. The plates were washed three times with PBST, and horseradish peroxidase (HRP)-conjugated goat anti-mouse antibodies (1: 10000) were added and incubated at 37°C for 1 h. ELISA plates were washed three times with PBST, and TMB substrate chromogenic solution was added and incubated in dark at room temperature for 3 min. The absorbance at 450 nm was detected by a multifunctional microplate reader (18).

### 2.5 Detection of lymphocyte proliferation

Lymphocyte proliferation was detected by MTS. All mice in immunogenicity experiments were sacrificed two weeks after the final immunization, and lymphocytes were dissociated from spleen tissues. The spleen tissues were filtered through a 70-μm cell sieve (Falcon, USA) to obtain single-cell suspensions, and 5 mL of Lympholyte^®^-M Cell Separation Media (CEDARLANE Canada) was added. Lymphocytes were isolated by centrifugation (2000 g, 20 min, a=0, 4°C). After centrifugation, the liquid was separated into three layers (plasma, lymphocyte and red blood cell) from top to bottom, and lymphocytes were transferred to another tube and washed three times with serum-free RPMI-1640 medium. The lymphocyte suspension (1×10^6^) mixed with vaccine polypeptide was added to 96-well plates (200 μL/well) and incubated at 37°C for 60 h in 5% CO_2_. Next, MTS was added (20 μL/well) and incubated at 37°C for 4 h in 5% CO_2_. Finally, the absorbance at 490 nm was detected by a multifunctional microplate reader (19).

### 2.6 Detection of cytokines

Mice in GILE and PBS groups were sacrificed two weeks after the final immunization and lymphocytes were prepared as described in Section 2.5. The levels of interleukin-4 (IL-4) and interferon-gamma (IFN-γ) were detected using the ELISpot Kit (Mabtech, Sweden) according to the manufacturer’s instructions. The ELISpot plates were washed four times with sterile 1×PBS (200 μL/well), and RPM1 1640 medium with 10% fetal bovine serum was added (200 μL/well) and incubated for 1 h. Then, plates were emptied and the splenocyte suspension (2.5×10^6^) mixed with vaccine polypeptide (10 μg/mL) was added (100 μL/well) and incubated for 48 h at 37°C in 5% CO_2_. Then, streptaviclin-ALP was added (100 μL/well) and incubated in dark at room temperature for 1 h. The plates were washed four times with PBS, and BCIP/NBT-Plus was added (100 μL/well). Finally, the spots were counted using an ELISPOT automatic platereader (AID Elispot Reader, AID, Germany).

FCM was used to verify the detection of cytokines. The lymphocytes suspension (1×10^6^) mixed with vaccine polypeptide (10 μg/mL) was added to 24-well plates (1 mL/well) and incubated for 0.5 h at 37°C in 5% CO_2_. The same amount of PBS and Cell Stimulation Cocktail (Invitrogen, USA) were added as negative control and positive control, respectively. Then, Brefeldin A Solution was added and incubated for 5.5 h at 37°C in 5% CO_2_. After that, Anti-Mouse CD4 PE-Cyanine7 (0.2 mg/mL) was added and incubated in dark for 15 min at room temperature. The supernatant was removed by centrifugation (4°C,1500 r/min,10 min). The Fix/Perm solution was added and incubated in dark for 30 min at room temperature. After centrifugation (4°C,500 g,10 min), 100 μL of 1×Permeabilization Buffer mixed with 0.5μL of Anti-Mouse IL-4 APC (0.5 mg/mL) and 0.2 μL of Anti-Mouse IFN-γ FITC (0.2 mg/mL) was added and incubated in dark for 30 min at room temperature. The supernatant was removed by centrifugation (4°C, 500 g, 10 min). Lymphocytes were washed three times with PBS and then re-suspended with PBS (500 μL). Finally, the fluorescence was detected by FCM (FACS AsiaIII^®^, ty20204251).

### 2.7 Evaluation of the protective effect of GILE

To evaluate the protective effect of GILE, the growth of cysts in mice livers were detected by Ultrasound (FUJIFILM Vevo^®^ Ultrasonic imager for small animals). Mice were anesthetized with 1.5% isoflurane in O_2_ and placed on a specially designed heated bed. After testing, mice were sacrificed, and their cysts were isolated and weighed.

### 2.8 Detection of CD4^+^, CD8^+^ T cells

Firstly, we used Lympholyte-M (CEDARLANE Canada) to separate and get all lymphocytes from mice spleen cell suspensions. Then, the lymphocytes we obtained were counted by Millirore PHCC60050 lymphocytes counter to make sure the lymphocytes number were 1×10^6^/mL. Next, the lymphocytes specific antibodies were used to label lymphocytes: the CD4^+^ T cells were labeled with Anti-Mouse CD4 FITC (Abcam) and CD8^+^ T cells were labeled with Anti-Mouse CD8a APC(Abcam) and at last CD4^+^, CD8^+^ T cells population level were detected by flow cytometry.

Mice in GILE and PBS groups of immune protection experiments were sacrificed, and lymphocytes were prepared as described in Section 2.5. T-Cells were detected by FCM. Lymphocytes were diluted (1×10^6^/uL) and cultured with Anti-Mouse CD8a APC (0.2 mg/mL) and Anti-Mouse CD4 FITC (0.5 mg/mL) in dark for 30 min at room temperature. After that, splenocytes were washed three times with PBS and re-suspended in 500 μL of PBS. Finally, the fluorescence was detected by FCM (FACS AsiaIII^®^, ty20204251).

### 2.9 Statistical analyses

All statistical analyses were performed using SPSS19.0 software and plots were drawn using GraphPad Prism8.0.1. Data were expressed as mean ± standard deviation. Comparisons between two groups were performed using an independent sample *t* test. P<0.05 indicated statistically significant difference (* P<0.05; ** P<0.01; *** P<0.001; ns no statistical significance).

## 3 Results

### 3.1 Construction of GILE

**Figure 1:**
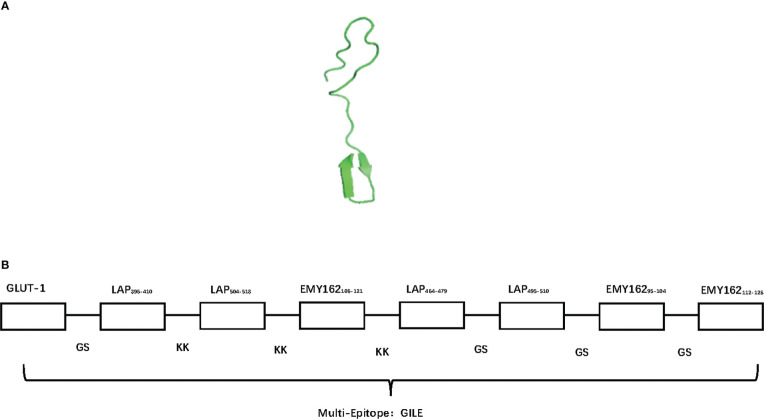
The bioinformatics construction of GILE results. **(A)**: The different homological models of GILE 3D construction of simulation by SWISS-MODEL; **(B)**: The order of epitopes in construction of GILE, signal T cell epitopes link by KK (Lysine- Lysine), signal B cell epitopes link by GS (Glycine-Serine).

The homological models of GILE were constructed by SWISS MODEL, and the model with good stretch and no mutual interference between epitopes was selected (**Figure 1A**). The order of epitopes in GILE was GLUT-1GSLAP_396-410_KKLAP_396-410_KKLAP_504-518_KKLAP_504-518_KKEMY_162106-121_KKEMY162_106-121_KKEMY162_106-121_KKLAP_464-479_GSLAP_464-479_GSLAP_495-510_GSLAP_495-510_GS EMY162_106-121_GSEMY162_106-121_GSEMY162_95-104_GSEMY162_95-104_GSEMY162_112-126_GSEMY162_112-126_ (**Figure 1B**). The secondary structure of GILE was predicted online using SOPMA, which consisted of α helix (13.84%), β fold (26.25%), β turn (14.08%), and random coil (45.82%). GILE might have good immunogenicity because of the presence of more β turn and random coil. The VMD results showed that GILE was a hydrophilic protein.

### 3.2 Expression and purification of proteins

**Figure 2:**
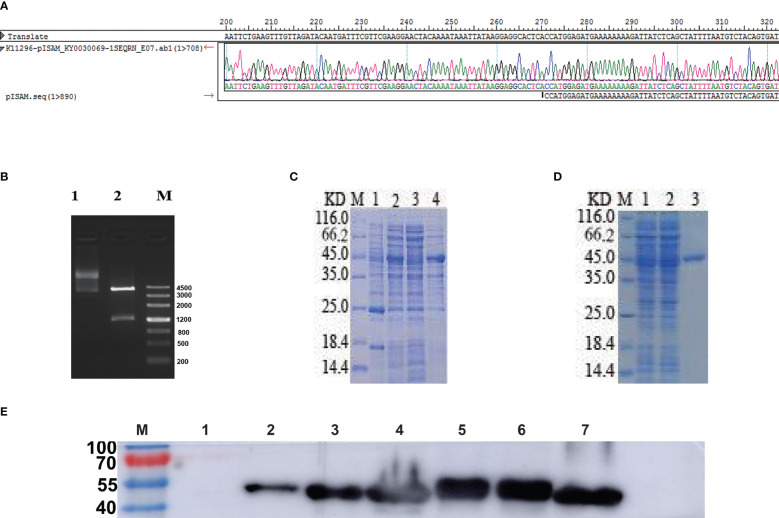
Identification and expression of GILE proteins. **(A)**: Observed and expected sequences of pCzn1-GILE. **(B)**: Enzyme digestion of pCzn1-GILE (M: Mark; Line1: before enzyme digestion; Line2: after enzyme digestion). **(C)**: Identification of GILE protein expression by SDS-PAGE (M: Mark, Lane 1: uninduced Escherichia coli expressing GILE proteins, Lane 2: induced Escherichia coli expressing GILE proteins, Lane 3: supernatant inducted with IPTG, Lane 4: precipitate inducted with IPTG). **(D)**: SDS-PAGE results of GILE protein purification (M: Mark, Line 1: samples after crushing, Line 2: samples discharged in purification, Lane 3: Purification). **(E)**: Western blot results (Lane 1: uninduced Escherichia coli expressing GILE proteins; Lane 2: induced Escherichia coli expressing GILE proteins; Lane 3: supernatant of induction with IPTG; Lane 4: precipitation of induction with IPTG; Lane 5: supernatant of inclusion-body protein dissolute in 8 m urea; Lane 6: precipitation of inclusion-body protein dissolute in 8 m urea; Lane 7: Purification).

The GILE gene was combined with N-6*His marker and connected to *pCzn1*, and the resulting recombinant plasmid pCzn1-*GILE* was identified by enzyme digestion and sequencing. pCzn1-*GILE* was transformed into *Escherichia coli* TOP10 strain and positive strain was selected for sequencing. The sequence alignment results showed a good agreement between observed and expected sequences of pCzn1-GILE. The target sequence *GILE* was successfully inserted into the plasmid (**Figure 2A**), and a bright band was clearly observed at 1266 bp after enzyme digestion (**Figure 2B**). *pCzn1-GILE* was transformed into Arctic Express cells to construct the prokaryotic expression system. After IPTG induction, the expression level of GILE proteins increased significantly and GILE was expressed in both supernatant and inclusion bodies (**Figure 2C**). The SDS-PAGE (**Figure 2D**) and western blot (**Figure 2E**) results showed that GILE proteins were successfully purified (44.68 KD).

### 3.3 Evaluation of immunogenicity

#### 3.3.1 Immunogenicity of GILE

**Figure 3:**
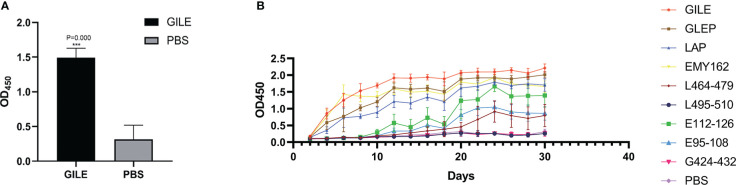
Detection of mice blood peripheral serum antibodies after inoculate vaccine by ELISA: **(A)**: The blood peripheral serum IgG levels of GILE and PBS groups after inoculate vaccine. **(B)**: Variation of blood peripheral serum IgG levels in 40 Days after inoculate vaccine. ***p<0.001.

Mice were sacrificed two weeks after the final immunization and their serum was isolated for detection of IgG by indirect ELISA. Compared to the PBS group, mice immunized with GILE exhibited a stronger humoral immune response (**Figure 3A**) and a significantly higher level of serum specific IgG (1.4947 ± 0.134 vs. 0.314 ± 0.204; *P* = 0.000). The dynamic changes of IgG were detected by indirect ELISA (**Figure 3B**). Compared to the PBS group, the serum of mice immunized with GILE could react with various peptides and the specific IgG level was increased gradually. Notably, the reaction with GILE proteins reached its maximum at the final immunization (OD450 nm=2.406). However, the IgG levels of the PBS group remained low throughout the experiment.

#### 3.3.2 Detection of spleen lymphocyte proliferation by MTS

**Figure 4:**
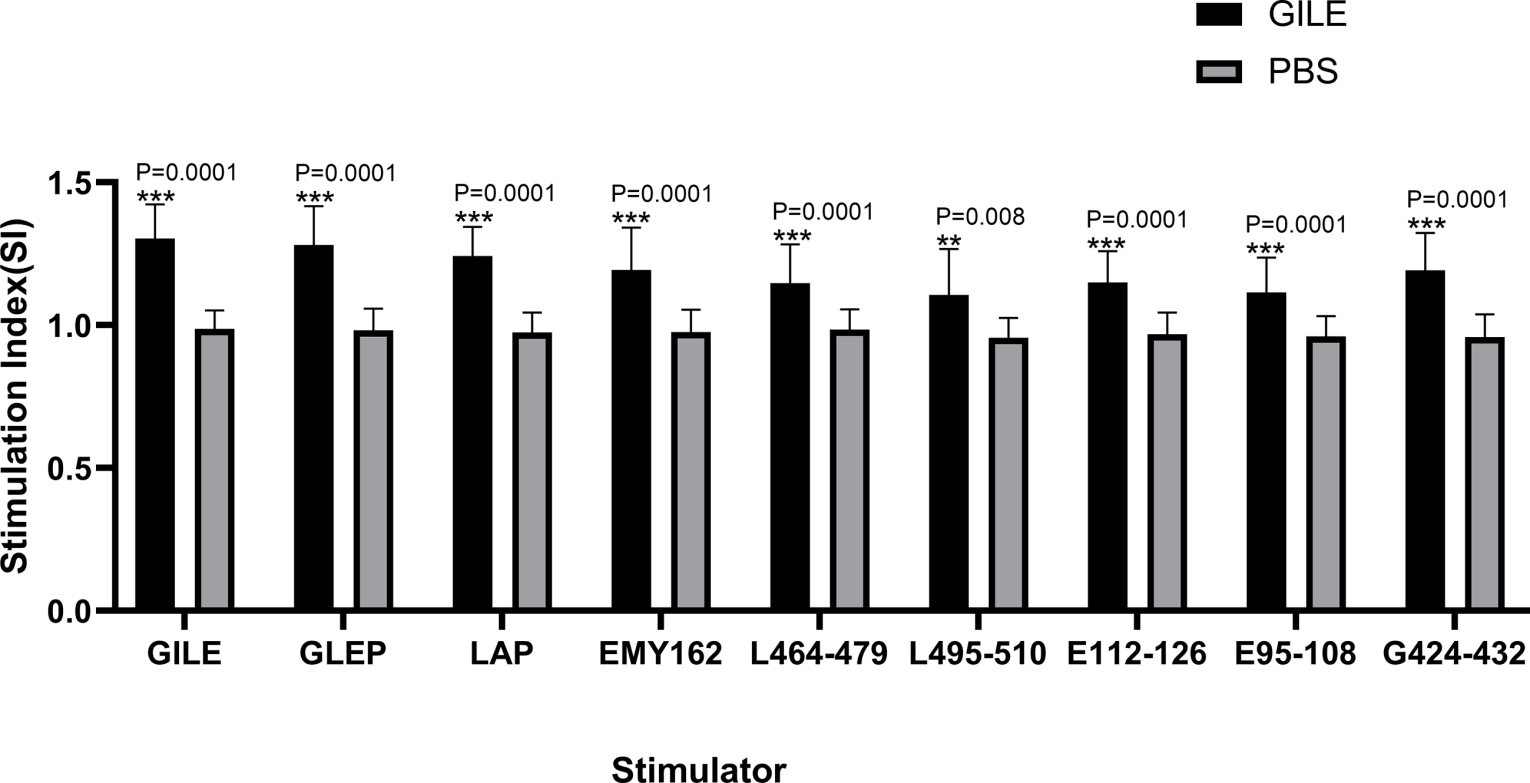
Detection of every group’s spleen lymphocyte proliferation by MTS the OD was 490nm. **p<0.01, ***p<0.001.

Spleen lymphocyte proliferation was determined by MTS, and the proteins and peptides in GILE were added to stimulate the proliferation. The GILE group showed higher responses than the PBS group (**Figure 4**). The lymphocytes of mice immunized with GILE could react with GILE recombinant proteins and epitope peptides. These results showed that immunization with GILE could induce cellular immunity to some proteins of *E. multilocularis* in mice.

#### 3.3.3 Detection of cytokines by ELISpot and FCM

**Figure 5:**
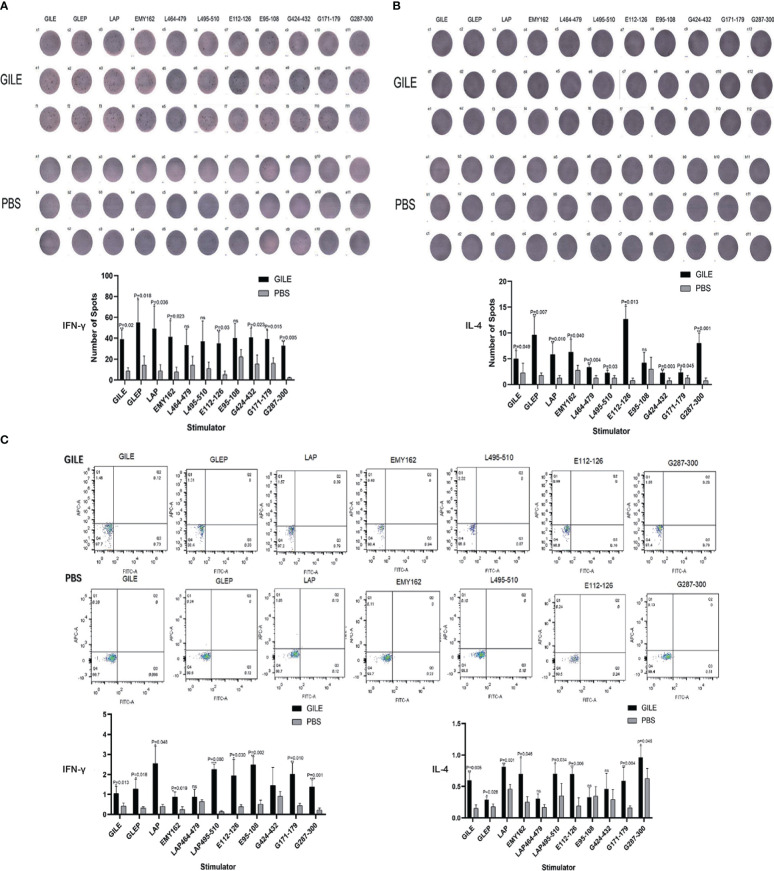
Detection of every group’s spleen cytokine by Elisapot and FCM. **(A)**: Detection of mice spleen IFN-γ level and counting every well plates spots number; **(B)**: Detection of mice spleen IL-4 level and counting every well plates spots number; **(C)**: Detection of cytokines IL-4 and IFN-γ level by FCM. ns means no statistical significance.

The proteins and peptides in GILE were used to stimulate spleen lymphocytes to secrete cytokines. The Th1-type cytokine IFN-γ and the Th2-type cytokine IL-4 were detected by ELISpot (**Figures 5A, B**) and FCM (**Figure 5C**), respectively. The levels of IL-4 and IFN-γ were higher in the GILE group, but no significant changes were observed in some cytokines stimulated by B epitope antigen.

### 3.4 Protective effect of GILE

#### 3.4.1 The growth and size of *Echinococcus multilocularis* cysts

**Figure 6:**
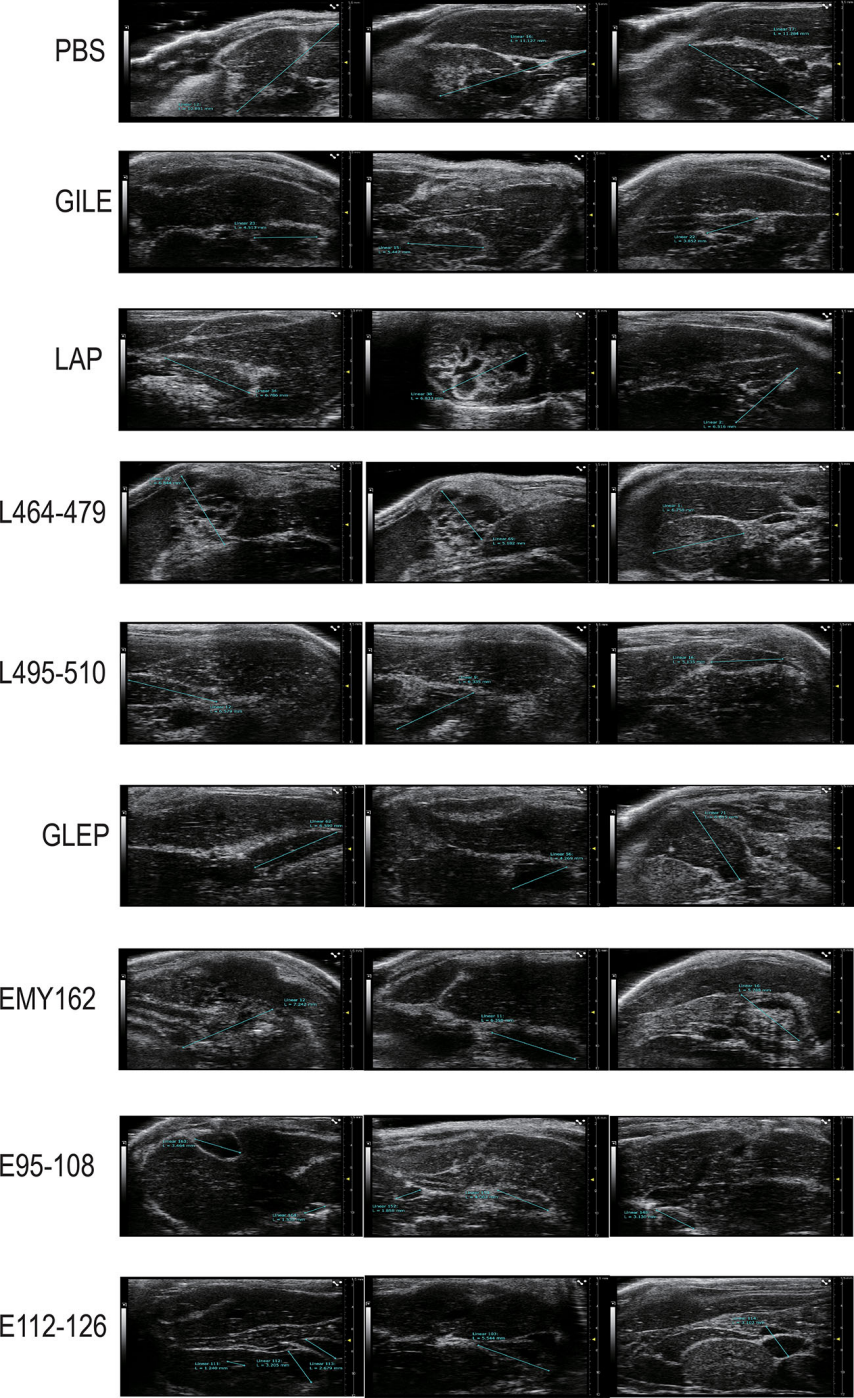
Detection of every challenge experiment mouse (PBS group and Vaccine groups) enterocoelia and liver cyst size by Ultrasound.

**Figure 7:**
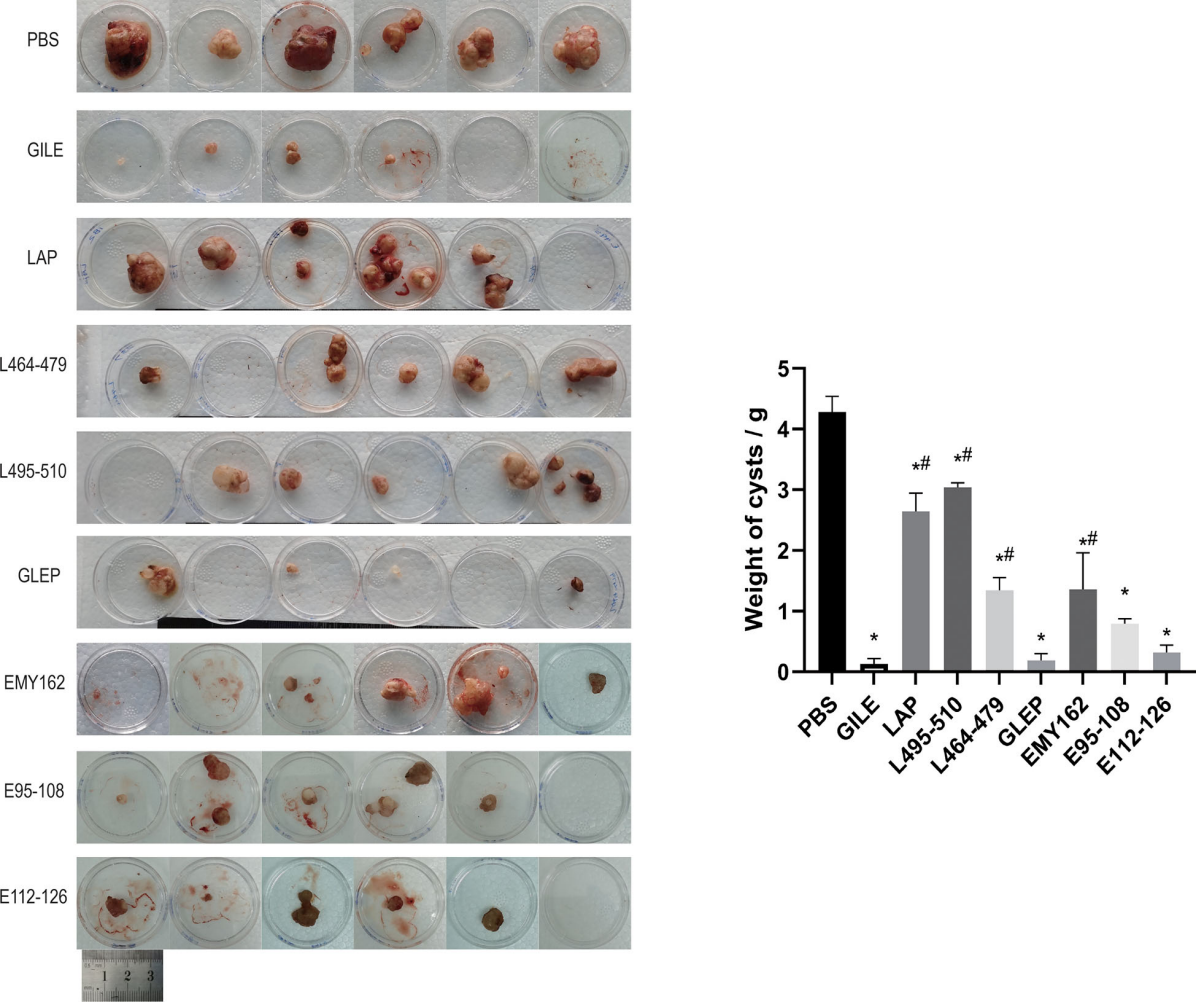
Detection of every challenge experiment mouse the size and number of cysts in enterocoelia and liver after prophylaxis (*: P<0.05 vs PBS, #: P<0.05 vs GILE).

The growth of hepatic cysts was evaluated by Ultrasound, and the results were shown in **Figure 6** and **Table 1**. The cysts of the GILE group were much smaller than those of protein and epitope groups, indicating that GILE with multiple dominant epitopes had a better protective effect against *E. multilocularis* infection. Cysts were isolated from the thoracic, abdominal, and subcutaneous tissues and weighed. As shown in **Figure 7**, the cysts of the GILE group (0.133 ± 0.083) were lighter compared to PBS (4.283 ± 0.258; *P* = 0.000), LAP (2.642 ± 0.300; *P* = 0.000), LAP_464-479_ (1.345 ± 0.206; *P* = 0.007), LAP_495-510_ (3.340 ± 1.074; *P* = 0.001), GLEP (0.188 ± 0.010; ns), EMY162 (1.360 ± 0.600; *P* = 0.010), EMY162 95-108 (0.793 ± 0.081; *P* = 0.012), and EMY162 _112-126_ (0.320 ± 0.118; ns) groups, suggesting that GILE might be effective in protecting against *E. multilocularis* infection.

**Table 1:** Maximum diameters of cysts (mm).

Groups	Diameters
PBS	9.76±2.38
GILE	4.88±0.77*
LAP	5.85±1.76*
LAP_495-510_	5.61±1.59*
LAP_464-479_	6.3±1.96*^#^
EMY162	7.57±1.63*^#^
EMY162_95-108_	5.35±0.86*
EMY162_112-126_	5.53±0.87*
GLEP	6.08±1.55*

(*: P<0.05 vs PBS, #: P<0.05 vs GILE).

#### 3.4.2 Detection of CD4^+^ and CD8^+^ T-Cell by FCM

**Figure 8:**
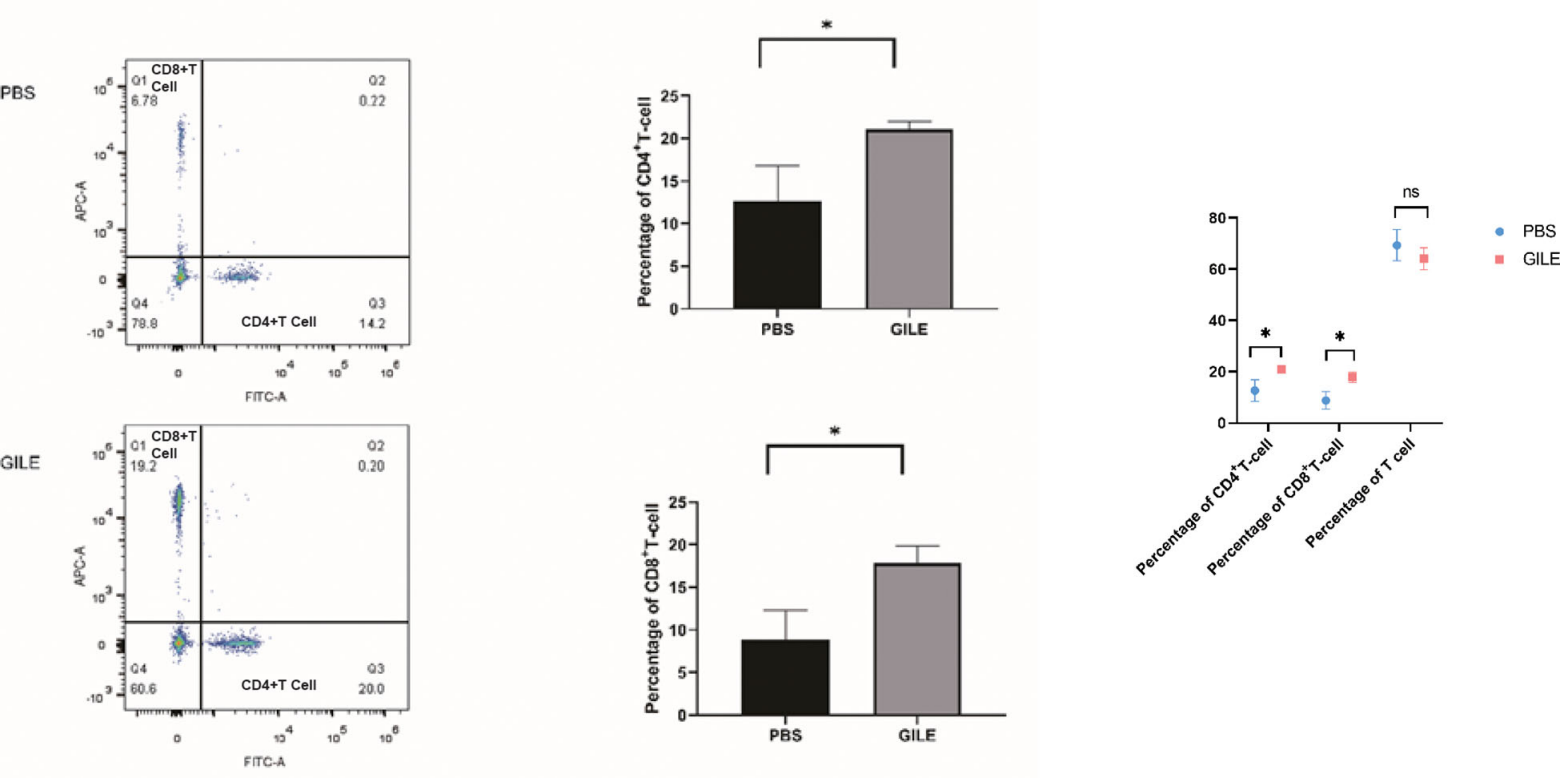
Detection of every groups’ mice spleen CD4^+^T cell and CD8^+^ T-Cell level by FCM; The CD4^+^ T cells were labeled with Anti-Mouse CD4 FITC (Abcam) and CD8^+^ T cells were labeled with Anti-Mouse CD8a APC(Abcam), CD4^+^, CD8^+^ T cells population level were detected by flow cytometry, Q1 showed the CD8^+^ cell population, Q3 showed the CD4^+^ cell population, Q4 showed the total lymphocyte population (*:P<0.05). ns means no statistical significance.

CD4^+^ and CD8^+^ T-Cell was detected by FCM. As shown in **Figure 8**, the number of CD4^+^ and CD8^+^ T-Cell of the GILE group was higher than that of the PBS group (CD4^+^T cell level: 12.667 ± 4.140 vs. 21.057 ± 0.958; *P*=0.026; CD8^+^ T cell level: 8.843 ± 3.434 vs. 17.853 ± 2.024; *P* = 0.027), suggesting that GILE could stimulate the production of CD4^+^ and CD8^+^ T-Cell to improve the immune function. There was no significant difference in lymphocyte count between PSB and GILE groups (69.33± 6.01 vs. GILE: 64.1± 4.25, *p*>0.05) (**Figure 8**).

## 4 Discussion

AE is one of the most life-threatening parasitic diseases caused by the metacestodes of *E. multilocularis*. However, a single protein vaccine might not provide adequate protection against *E. multilocularis* infection because different types and amounts of proteins would be expressed in different developmental stages (egg, protoscolece and adult tapeworm) of *E. multilocularis*. In this study, a multi-epitope vaccine GILE has been successfully constructed based on the dominant epitopes of EmEMY162, EmLAP and EmGLUT1 identified in our previous studies (**Figures 1**, **2**). The immunogenicity experiments have shown that GILE has good antigenicity (**Figures 3**, **4**, **5**). EMY162 plays a key role in the growth of *E. multilocularis* and it is expressed in all developmental stages of *E. multilocularis*. Thus, it may induce host immune responses in each developmental stage of *E. multilocularis* (20). Our previous study has also suggested that the recombinant *E. multilocularis* EMY162 (rEmEMY162) could protect against *E. multilocularis* infection in mice (74.3%) (10). LAP is involved in the nutrient absorption of the parasite and its invasion in the host, and it can prevent not only the infection of *E. multilocularis* infection but also its metabolism and infiltration. The recombinant *E. multilocularis* LAP can significantly reduce the number and size of cysts in mice infected with *E. multilocularis* (9). GLUT is related to glucose absorption and energy supply in most parasites*. E. multilocularis* has two GLUT genotypes (GLUT1 and GLUT2), and only GLUT1 has high glucose transport activity (21). To sum up, EmEMY162, EmLAP and EmGLUT1 play important roles in the survival and development of *E. multiloculari*s in the host.

In the design of GILE, GS and KK amino acid sequences were introduced to ensure the stability between epitopes, inhibit the formation of new epitopes, maintain the antigenic specificity of epitopes, and improve the efficiency of antigen-presenting cells (22). Because of the low and unstable immunogenicity of a single epitope (23), epitopes were repeated two to three times in order to enhance the immunogenicity and stability of GILE. In this study, the highly immunogenic B cell epitope of EmMY162 was placed at the end to retain the immunogenicity of each epitope as much as possible (21) (**Figure 1B**).

The proportion of α helix, β fold, β turn and random coil is 13.84%, 26.25%, 14.88% and 45.82% in the secondary structure of GILE, respectively. The stability of α helix and β fold is dependent on hydrogen bonds and they are located in the interior of the protein. β turn and random coil are the main recognition regions of leukocytes and antibodies (24). GILE can be easily recognized by leukocytes and antibodies because of the presence of β turn and random coil. GILE might have good immunogenicity. The Swiss-model analysis has shown that the tertiary structure of GILE is extensible no shielding or interference between epitopes, which contributes to the combination of epitopes in GILE with antigen presenting cells and effector cells to induce immune responses (**Figure 1A**). The VMD analysis has shown that GILE is a hydrophilic protein.

The animal model was established by intraperitoneal injection of protoscoleces in this study, which is consistent with previous studies (25). However, it should be noted that this differs substantially from natural infection because the failure rate is higher than that of natural infection, and most *E. multilocularis* grows in the abdominal cavity and liver rather than in a single organ.

In this study, it is found that the multi-epitope vaccine GILE has better protective effect than single epitopes and proteins (**Figures 6**, **7**; **Table 1**). The cysts are significantly smaller in the GILE group. The same trend is observed for the weight and size of cysts in the abdominal cavity. The multiple-epitope vaccine is more effective in inducing specific immune responses than single epitopes and proteins. Thus, the dominant epitopes of EmEMY162, EmLAP and EmGLUT1 identified in our previous studies are used to construct the multiple-epitope vaccine GILE. The structure of GILE was optimized to make it more hydrophilic and have more β turn and random coil. As a result, it is easier for GLIE to contact antigen presenting cells and activate immune cells to produce antibodies.

The antibody-dependent cell-mediated cytotoxicity (ADCC) is involved in the immunity of the host against early infection. Antibodies play a key role in the immune response against cysticercus taenia (26), and IgG acts as a protective antibody in humoral immunity. In this study, it is found that immunization with GILE induces a strong specific humoral immune response in BALB/C mice (**Figures 3**, **8**). High levels of IgG are observed in the serum of mice immunized with GILE, and the serum could react specifically with various peptides and proteins in GILE. The IgG level increases gradually and reach a maximum at week 4 of immunization with GILE, but it remains low in the PBS group throughout the experiment. Thus, it is concluded that GILE could induce strong humoral immune responses and has good immunogenicity.

Th1/Th2 balance is critical during the infection with parasites such as trypanosoma (27), schistosoma (28), and echinococcus (29). There are several strategies to evade host immune attack in *E. multilocularis* infection, and the immune escape mechanism involves the transition from the protective immune (Th1) response to the non-protective immune (Th2) response (26). Th1-polarized cytokines could kill cysticerci in the early stage of infection and the immune response is gradually transformed into Th2 response during the chronic stage (30). IL-12 could induce Th1 immunity and the secretion of IL-2, TNF, and IFN-γ, which results in recruitment and activation of immune cells (31). The Th1 immunity is gradually changed into the Th2 immunity because of the increase of IL-10, which is associated with immune regulation and plays a role in limiting inflammation (32). In mice infected with *E. multilocularis*, the immune response is dominated by Th1 cells in the early stage but by Th2 cells in the later stage (26). The transition from Th1- to Th2-dominated immune response may account for why *E. multilocularis* could evade the surveillance of the immune system (33). The levels of IFN-γ and IL-4 are significantly increased in mice immunized with GILE compared to the PBS group (**Figures 4**, **5**), suggesting that GILE might inhibit the development of *E. multilocularis* by inducing Th1-Th2 mixed immune response. Compared to the PSB group, both CD4^+^T cell and CD8^+^ T cell were increased in the GILE group (**Figure 8**). In the host infected by parasites, CD4^+^ cells may be first activated to release cytokines such as IL-2, which in turn stimulate CD8^+^ cells that can exert their effects through direct cytotoxicity or secretion of cytokines (34). Th1/Th2 shift as a side reaction of *E. Multilocularis* infection can lead to parasite immune evasion and even hypersensitivity (35). Th1/Th2 imbalance takes place in the host infected with *E. Multilocularis* and the shift between Th1 and Th2 requires the transition of the intermediate form Th0. IFN-α, TNF-β and IL-12 secreted by macrophages and NK cells could shift allergen specific T cells from Th2/Th0 to Th0/Th1; while IL-4 and anti-IL-12 mAb could induce T cells to shift from Th1 to Th0/Th2. Both Th1 and Th2 cells can express the β1 chain of the IL-12 receptor, but only Th1 cells can express the β2 chain. Th2 cells without β2 chain could not be directly transformed into Th1 cells (36), but Th1 cells could be transformed into Th2 cells (37). These results indicate that the shift from Th1 to Th2 induced by *E. Multilocularis* infection would cause immunosuppression of the host and thus allow *E. Multilocularis* to escape from the immune system and continue to grow. Therefore, the dominant epitopes of Th1 and Th2 were added in constructing GILE to avoid Th1/Th2 shift, and Freund’s adjuvant that could activate both Th1 and Th2 cells were used to make the immune response more balanced and specific and partially avoid Th1/Th2 shift (38). However, as Freund’s adjuvants are not approved for veterinary or human use due to high reactogenicity, we have to choose commercial adjuvant that has similar effects as Freund’s adjuvants but low reactogenicity for future clinical trials.

GILE has been found to be better than single protein vaccines such as 14-3-3, EMY162, Em95 (13). GILE contains a variety of identified epitopes that can more effectively reduce the size and growth of cysts and induce antibody secretion This multi-epitope vaccine has Th1/Th2 mixed epitope that can improve immunologic balance, avoid Th1/Th2 shift, reduce hypersensitivity and parasite immune escape. Ultrasound was performed to evaluate the growth of hepatic cysts in mice. A significant difference is found in the maximum diameter of liver cysts between GILE and control groups. The cysts weight results also reveal that the multi-epitope vaccine GILE could better protect mice from *E. multilocularis* infection than single protein vaccines such as 14-3-3, EMY162, and Em95. GILE might induce stronger immune responses compared to the single epitope or protein vaccine. It protects the host against *E. multilocularis* infection by effectively activating the immune system and inducing stronger immune responses. Compared to the PBS group, GILE induces an increase in the percentage of CD4^+^ and CD8^+^ T cells. It is interesting that the percentage of lymphocytes is not increased, but that of activated lymphocytes (CD4^+^ and CD8^+^ T cells) is increased. The same phenomenon is observed for *E.* granulosus, the Eg95-EgA31 fusion gene alfalfa to induce immunity vaccination, mice spleen T cell proliferation inhibition of splenic cells apoptosis, enable higher CD4 ^+^ T cell/CD8 ^+^T cell ratio, but whole lymphocytes level was not increased (39). Thus, GILE can effectively activate immunocompetence. In this study we used flow cytometry to evaluate the CD4^+^T cell and CD8^+^T population following the immunization of SPF mouse with vaccination, but we didn’t evaluate the total T cell level by flow cytometry, because on one hand we used Lympholyte-M to get all lymphocytes from mice spleen cell suspensions. Then, the lymphocytes we obtained were counted by Millirore PHCC60050 cell counter to ensure the lymphocytes number were 1×10^6^. Next, we use the specific antibodies to label lymphocytes: The CD4^+^ T cells were labeled with Anti-Mouse CD4 FITC (Abcam) and CD8^+^ T cells were labeled with Anti-Mouse CD8a APC(Abcam). At last, CD4^+^, CD8^+^ T cells population level were detected by flow cytometry.So, at the beginning we identified the total number of lymphocytes by cell counter, then the CD8a APC and CD4 FITC were used to confirm the activated lymphocyte proportion level. Therefore, we can preliminarily measure the level of effector T cells in this study and confirm the immune effect of GILE.

In this study, we found increased levels of CD4+ T cells and CD8+ T cells in both the PBS and GILE groups, but increased levels of CD8+ T cells were very rare. Because the E. Multilocularis was extracellular infection and activated the humoral immunity, so the CD8+ T cell was one of nonspecific T cells in E. Multilocularis infection. The other research also reported the non-specific CD8+ T cells increase during parasitism and play a role in protective immunity against parasites (40).

But there still have some deficiencies in this study. Firstly, we only detected CD4^+^, CD8^+^T cell levels after inoculate vaccine, but it was lack of additional activate T cell level and the dynamically CD4^+^, CD8^+^ T cell situation, another improvement of our experiment is to monitor the dynamically variety of cytokine and T cell parameters including but not limited to detected T cell costimulatory molecules, Th9 cell and Treg by Elisa and FCM after the inoculated. In further study, we will systematically evaluate the all aspects of vaccine.

## 5 Conclusions

In this study, a multi-epitope vaccine GILE has been successfully constructed to against *E. multilocularis* infection in BALB/C mice. The immune protection effect of GILE could be mediated by inducing the production of specific serum antibodies, maintaining the Th1-Th2 mixed immune response, produced the CD4^+^, CD8^+^ T cell and improving the immune eliminate function of the host.

## Data availability statement

The datasets presented in this study can be found in online repositories. The names of the repository/repositories and accession number(s) can be found in the article/supplementary material.

## Ethics statement

All animal experiments were performed in compliance with the regulations of the Ministry of Science and Technology of China and approved by the Experimental Committee of Qinghai University (QHDX-2018-09).

## Author contributions

PZ: Writing - original draft, Writing - review & editing, Visualization. ZZ: Writing MH: Investigation. LW: Investigation, Supervision. LF: Investigation,Supervision. YX: Investigation. YD: Conceptualization, Project administration. XM: Conceptualization, Project administration. FT: Writing - review & editing, Methodology. RL: Writing - review & editing, Methodology. All authors contributed to the article and approved the submitted version.
